# Intramuscular hemangioma in the zygomaticus muscle: A rare case report presentation and diagnosis

**DOI:** 10.1016/j.ijscr.2020.07.068

**Published:** 2020-07-28

**Authors:** Ashwag Yagoub Aloyouny, Mohamed Saleh Mehanny, Hamad Nasser Albagieh, Wafa Mohammed Alfaleh, Soad Mahmoud Mansour, Fahmy A. Mobarak

**Affiliations:** a Basic Dental Sciences, College of Dentistry, Princess Nourah Bint Abdulrahman University, Riyadh, Saudi Arabia; b Oral Medicine and Diagnostic Science Department, College of Dentistry, King Saud University, Riyadh, Saudi Arabia; c Oral and Maxillofacial Surgery, Surgery Department, Faculty of Dentistry, Cairo University, Cairo, Egypt

**Keywords:** Case reports, Intramuscular hemangioma, Vascular lesions, Zygomatic bone, Zygomaticus muscle

## Abstract

**Introduction:**

intramuscular hemangioma (IMH) is a relatively rare benign vascular tumor of the skeletal muscles. It shows less than 1 % of all soft tissue hemangiomas. In the head and neck area, it occurs usually in the masseter muscle followed by temporalis and sternocleidomastoid muscles.

**Presentation of case:**

we present a case of 25-year-old male patient with a chief complaint of slowly growing facial swelling in the left zygomatic area. Clinical, imaging and histopathological evaluation lead to the diagnosis of intramuscular hemangioma in the zygomaticus major muscle.

**Discussion:**

IMH in the zygomaticus muscle is very rare; hence, the clinical diagnosis of IMH is challenging. Different diagnostic procedures can be used such as CT and MRI. In addition, the ideal therapy for esthetic disfiguring IMH in the head are is the complete surgical excision of the lesion. Through the review of literature and to our knowledge this case is the first report of intramuscular hemangioma in the zygomaticus muscle.

**Conclusion:**

IMHs are rare in the head and neck area and must be considered in differential diagnosis of isolated muscle mass in this region.

## Highlights

• Intramuscular hemangioma is a relatively rare benign vascular tumor of the skeletal muscles.
• The initial diagnosis of Intramuscular hemangioma is complicated due to low incidence and variation of symptoms.
• Intramuscular hemangioma in the zygomaticus major muscle is extremely rare.

## 1 Introduction

Hemangiomas are neoplasms of endothelial origin; they are composed of vascular spaces emerging from endothelial cells. It commonly found in infants in the first year of life and then the lesion disappears with age [1]. Hemangioma commonly occur on the skin and subcutaneous tissues. Dissimilar to cutaneous hemangioma, intramuscular hemangioma (IMH), which presents less than 1 % of all soft tissue hemangioma [2], slowly grows in size and does not tend to disappear spontaneously. It is usually noted in the second and third decades of life. IMH is a vascular benign proliferation that mostly occur in the skeletal muscles of the lower limbs and rarely in the head and neck area [3]. The etiology of IMH is not clear yet, although trauma, abnormal embryonic tissue development, hormonal changes are considered predisposing factors in the growth of the intramuscular hemangioma by stimulating more blood flow in the mass [3,4]. Intramuscular hemangioma, usually manifested clinically as painless, slowly growing with clear boundaries of the mass, and the development of calcified thrombi, which known as phleboliths [5]. The initial diagnosis of IMH is complicated due to low incidence and variation of symptoms, most of the cases are misdiagnosed preoperatively [6]. Radiographic investigation including computed tomography and magnetic resonance imaging are important for the evaluation of the IMH. A case of capillary hemangioma located in the zygomaticus major muscle with multiple phleboliths in a 25 years old male is reported here. To our knowledge this case is the first report of intramuscular hemangioma in the zygomaticus major muscle. (Researchregistry5769) “The work has been reported in line with the SCARE criteria” [7].

## 2 Presentation of case

**Fig. 1:**
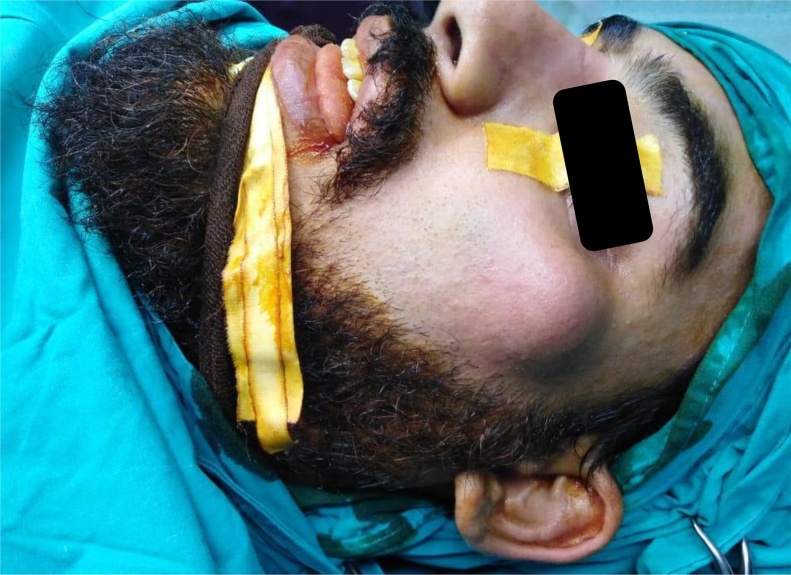
Pre-operative lateral view of left zygoma displaying obvious expansion.

**Fig. 2:**
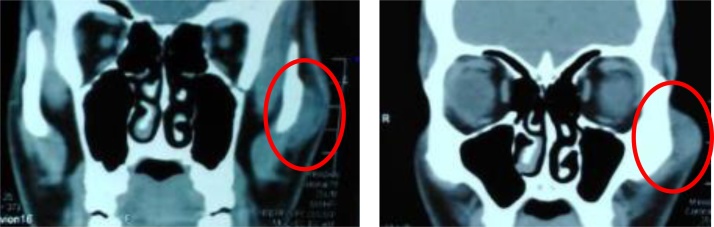
Coronal soft tissue CT scans obtained in 25 years-old Male: A circle shows heterogenous iso-density mass with discrete numerous low attenuation areas. A mass measuring 2.5 × 2 cm in size with well- contoured margin. which abutting on the left zygoma without radiographic signs of intra- bony involvement.

**Fig. 3:**
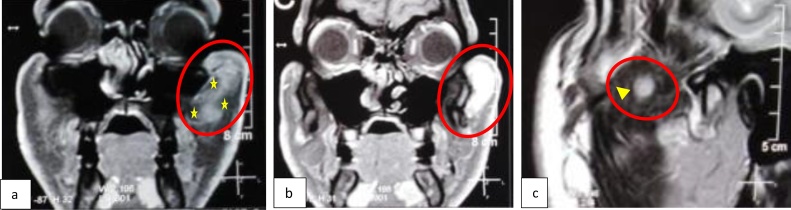
Contrast enhanced Magnetic Resonance Images; (a, b) Coronal PD images: circles show heterogenous well-Defined hyperintense soft tissue mass with scattered hypointense spaces suggesting phleboliths “star” and (c) Sagittal T2 image shows hyperintense mass with surrounded non-uniform hypointense fatty tissue “arrowhead”. The mass involved the zygomatic muscles and abutting on the left zygomatic bony complex.

A 25-year-old male patient referred to with no significant medical history presented with a chief complaint of slowly growing facial swelling in the left zygomatic area which had been gradually increasing in size for the last eight months. On clinical examination (Fig. 1), a smooth, painless, palpable and compressible, firm, soft tissue mass was observed in the left zygomatic area. The skin over the swelling was light red in color and no other abnormalities were observed. The patient denied any history of prior trauma or surgery to the area of swelling. Past medical history was non-significant. Moreover, He is not taking any prescribed or over the counter medications. The patient is not aware of any family medical history and genetic problems. Also, the patient denied the use of tobacco products. The patient reported that the lesion caused cosmetic disfiguring of his facial appearance which had a psychosocial impact on his life. Radiographic investigation including Computed tomography (Fig. 2) showed heterogenous iso-density mass with discrete numerous low attenuation areas. A mass measuring 2.5 × 2.0 cm in size with well-delineated margin, which abutting on the left zygoma without radiographic signs of intra-bony involvement. Further, Contrast Enhanced Magnetic Resonance Images (Fig. 3) showed heterogenous well-defined hyperintense soft tissue mass with scattered hypointense spaces suggesting phleboliths and non-uniform hypointense fatty tissue. The mass involved the zygomatic major muscle and abutting on the left zygomatic bone. (Researchregistry5769) https://www.researchregistry.com/register-now#user-researchregistry/.

**Fig. 4:**
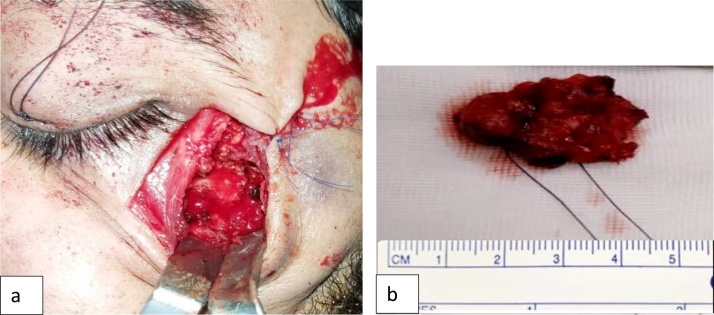
(a) Intraoperative surgical picture shows extended subciliary incision. (b) Post excisional macroscopic appearance of intramuscular mass measured 2.5 × 2 × 1.5 cm.

**Fig. 5:**
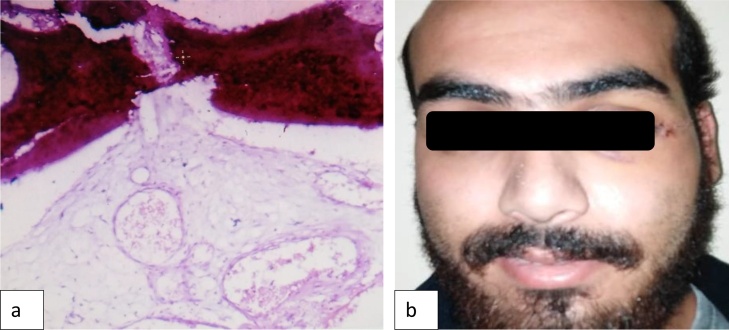
(a) Histopathological analysis shows lobulated tumor composed of spindle cells; likely endothelial cells with pericytes. (b) Post-operative presentation of patient.

In the present case, the patient was provided with information regarding the condition, possible treatment options and associated outcomes. Moreover, on consenting for surgical therapy under general anesthesia, the patient underwent complete excision of the intra-zygomaticus major muscle mass via an extended subciliary approach (Fig. 4a). The surgery was performed by a professor of oral and maxillofacial surgery. An excisional biopsy was performed, a single mass of tissue was obtained which was well-circumscribed with hemorrhagic areas, rubbery in consistency, reddish in color, measured 2.5 × 2.0 × 1.5 cm (Fig. 4b). Also, within in the same container, two flattened bony fragments, measured 1.0 × 1.0 cm. were sent for histopathology. Histopathologic evaluation of the lesion (Fig. 5a) revealed a lobulated tumor composed of spindle cells; likely endothelial cells with pericytes. The tumor involved the skeletal muscle tissue and fat still preserving the lobulated pattern. The histopathologic diagnosis revealed intramuscular capillary hemangioma. Noteworthy, post-surgical instructions were provided in verbal and written forms. Follow-up visit (Fig. 5b) revealed good healing with no complications in the area of zygomaticus major muscle. The patient tolerated the procedure well, recovered faster than expected, also, he was compliance with the post-surgical instructions. At two-year follow-up, the patient was lesion recurrence-free.

## 3 Discussion

Intramuscular hemangioma (IMH) was first reported in 1843 by Liston. IMH is comparatively rare benign vascular tumor of the skeletal muscles. It shows less than 1% of all soft tissue hemangiomas. IMH usually occurs in the large muscles of the trunk and extremities. In the head and neck area, it occurs usually in the masseter muscle followed by temporalis and sternocleidomastoid muscles [2]. Intramuscular hemangiomas are categorized histologically depending on the vessel size into capillary hemangioma (small vessels <140 mm), cavernous hemangioma (large vessels >140 mm), and venous hemangioma with an incidence of 68 %, 26 %, and 6 % of all IMH respectively [8]. Capillary hemangioma mostly characterized by a short history of symptoms and they are highly cellular which describe the firmness and deficiency in the clinical signs and symptoms. On the other hand, cavernous hemangioma usually present with longer history of symptoms, causes painful, larger lesions [3]. Around 15%–20% of Intramuscular hemangioma contain phleboliths or calcifications; caused by mineralized thrombi.

The etiology of IMH is unknown, although trauma, abnormal embryonic tissue development, hormonal changes are considered predisposing factors in the growth of the IMH by stimulating more blood flow in the mass [3,4]. Initially, IMH clinically occur as a painless, slowly growing mass before making any noticeable symptoms [1,3]. Most of the time, IMH is not noted until esthetic deformities, unanticipated enlargement, or pain occurs [6]. The differential diagnosis of a mass in the zygomatic area includes lipoma, dermoid cyst, neurofibroma, soft tissue sarcoma and leiomyoma. Due to low incidence, the location and variation of symptoms, most of the cases are misdiagnosed before surgery and histopathology evaluation, less than 8 % of the cases has accurate diagnosis preoperatively [6]. Fine needle aspiration biopsy of the lesion showed blood fluid; hence, it was positive for vascular lesion. Furthermore, radiographic investigation including computed tomography (CT) and magnetic resonance imaging (MRI) play an important role in the evaluation of the IMH. Contrast-enhanced CT plays an important role in the identification of the size and shape of the tumor. Also, calcification within the tumor or bone involvement in the pre-contrast scans can be evaluated. MRI is very important for the identification of tumor, it shows certain radiologic features that would suggest hemangioma; had isointense signals on T1-weighted sequences and high signals on T2-weighted sequences, and the phlebolith shows hypointense signal on T1/T2-weighted imaging. It is worth mentioning that angiography is one of the diagnostic aids for the vascular lesions; however, this type of diagnostic procedure was not needed because the MRI in this case showed a static-type vascular lesion with no feeder vessel, which meant the lesion was most probably a hemangioma. In contrast to the hemangiomas, vascular malformations are either arteriovenous malformations which are fast-flow-type vascular lesions or slow-flow-type vascular lesions which are classified as venous or capillary or lymphatic malformations [9]. Besides, the patient declined to go through angiography procedure due to some financial issues.

Treatment of the IMH should be customised based on the patient age, lesion site, accessibility, extent, growth rate, and esthetic considerations. All conservative treatments such as systemic corticosteroids, embolization, radiation, and sclerotherapy have high recurrence rate. Due to the close approximation of the lesion to a vital surrounding structure (the eye), the surgeon was against other conservative therapy options. Moreover, sclerotherapy causes sclerosis and fibrosis of the lesion and this material may pass through the inferior orbital artery and cause eye damage. Embolization was not recommended because the lesion as shown in the MRI did not have a feeder vessel.

The optimal treatment for small hemangioma in adults is the complete surgical excision of the lesion with a rim of normal surrounding muscle to minimize the risk of recurrence [10]. Capillary hemangioma, cavernous hemangioma, and venous hemangioma recurrence rates were 20 %, 9 %, and 28 % accordingly [11].

## 4 Conclusion

To sum up, intramuscular hemangiomas are rare in the head and neck area and should to be considered in differential diagnosis of isolated muscle mass in this region. Professional clinical examination, imaging interpretation and histopathological evaluation of the completely excised tumor gives an accurate final diagnosis of the intramuscular hemangiomas.

## Declaration of Competing Interest

The authors declare that they have no conflicts of interest.
